# Rejection sensitivity and symptom severity in patients with borderline personality disorder: effects of childhood maltreatment and self-esteem

**DOI:** 10.1186/s40479-015-0025-x

**Published:** 2015-03-20

**Authors:** Melanie Bungert, Lisa Liebke, Janine Thome, Katrin Haeussler, Martin Bohus, Stefanie Lis

**Affiliations:** Department of Psychosomatic Medicine and Psychotherapy, Central Institute of Mental Health Mannheim, Medical Faculty Mannheim/Heidelberg University, Germany, PO Box 12 21 20, 68072 Mannheim, Germany

**Keywords:** Borderline personality disorder, Rejection sensitivity, Childhood maltreatment, Self-esteem, Anxiety, Aggression

## Abstract

**Background:**

Interpersonal dysfunction in Borderline Personality Disorder (BPD) is characterized by an ‘anxious preoccupation with real or imagined abandonment’ (DSM-5). This symptom description bears a close resemblance to that of rejection sensitivity, a cognitive affective disposition that affects perceptions, emotions and behavior in the context of social rejection. The present study investigates the level of rejection sensitivity in acute and remitted BPD patients and its relation to BPD symptom severity, childhood maltreatment, and self-esteem.

**Methods:**

Data were obtained from 167 female subjects: 77 with acute BPD, 15 with remitted BPD, and 75 healthy controls who were matched with the patients for age and education. The instruments used for assessment were the Rejection Sensitivity Questionnaire, the short version of the Borderline Symptom List, the Childhood Trauma Questionnaire, and the Rosenberg Self-Esteem Scale.

**Results:**

Both acute and remitted BPD patients had higher scores on the Rejection Sensitivity Questionnaire than did healthy controls. Lower self-esteem was found to be positively correlated with both increased BPD symptom severity and higher rejection sensitivity, and mediated the relation between the two. History of childhood maltreatment did not correlate with rejection sensitivity, BPD symptom severity, or self-esteem.

**Conclusions:**

Our findings support the hypothesis that rejection sensitivity is an important component in BPD, even for remitted BPD patients. Level of self-esteem appears to be a relevant factor in the relationship between rejection sensitivity and BPD symptom severity. Therapeutic interventions for BPD would do well to target rejection sensitivity.

**Electronic supplementary material:**

The online version of this article (doi:10.1186/s40479-015-0025-x) contains supplementary material, which is available to authorized users.

## Background

One of the core symptom domains in borderline personality disorder (BPD) is interpersonal dysfunction. In the Diagnostic and Statistical Manual of Mental Disorders (DSM-5), one of the diagnostic criteria of such interpersonal dysfunction is ‘frantic efforts to avoid real or imagined abandonment’. In the alternative model of DSM-5, section III, this criterion has been reformulated as ‘anxious preoccupation with real or imagined abandonment’- a reformulation which shifts emphasis from alterations in overt behavior to alterations in cognition and affect. This symptom description bears a striking similarity to the social psychology concept of rejection sensitivity (RS), defined as the cognitive affective disposition that influences expectations, perceptions, and behavior within the context of social rejection [1]. Downey et al. [2] describe people high in rejection sensitivity as tending to ‘anxiously expect, readily perceive, and intensely react to rejection’. In a later publication, the authors extend the concept of RS by postulating a ‘defensive motivational system’ as underlying physiological mechanism, which enables an efficient way to detect and react to a potential threat of belonging [2,3]. They defined RS according to the general approach avoidance motivational model [4,5] that distinguishes two affective-motivational systems: an approach system responds to positive stimuli, while an avoidance/defensive system is responsive to negative stimuli and leads when activated to a preferred perception and processing of potential threat cues [4,5]. Downey et al. [2] propose that this defensive motivational system is particularly sensitive for social rejection in people high in RS. Experimental studies support this hypothesis by using the startle reflex, which is regarded to be a reliable indicator of the activation level of the defensive motivational system [5]. Downey et al. [2] observed enhanced startle reflex responses in people high in RS particularly while viewing rejection-related pictures compared to people low in RS and compared to pictures showing acceptance or non-interpersonal positive or negative scenes.

In the last few decades, a multitude of studies have investigated causes, consequences, and modulating factors of RS in healthy individuals by means of a self-report questionnaire, i.e. the Rejection Sensitivity Questionnaire (RSQ) developed by Downey et al. [2]. Early experiences of belonging or rejection have been shown to be crucial to the development of RS [6-10]. RS affects social functioning in different social domains: for example, in romantic partnerships, high RS predisposes an individual to perceive ambiguous or insensitive behavior of the partner as rejection [1]. In contrast to the hypersensitivity of individuals high in RS, low RS has been linked to an interpersonal optimism which might facilitate the initiation of social interactions, i.e. a positive bias towards a high expectancy of being accepted [11].

Several studies have suggested that high RS predisposes for the development of mental disorders such as social anxiety or depression (for review, see [12]), while Staebler et al. [13] underlined the relevance of RS particularly in the context of BPD. However, so far, only a few studies have investigated RS in small clinical samples of BPD patients. Staebler et al. [13] reported that both in-patients and outpatients with BPD had higher RS compared to either healthy individuals or outpatients with mood disorders, anxiety disorders, social anxiety disorder, and avoidant personality disorder. The finding of higher RS in BPD subjects compared to healthy controls has been confirmed by Berenson et al. [14] and Domsalla et al. [15]. All these studies had effect sizes between 1.8 and 3.2 (Cohen’s d), suggesting a high separation between healthy individuals and patients with BPD. Additionally, there is an increasing body of evidence linking RS to the number of borderline features in non-clinical samples [16-21]. These studies point to an important role of RS as a mediator in the relation of BPD features with for example anxious and avoidant attachment style and the appraisal of other individuals [16,18]. The link between RS and BPD features is especially pronounced in subjects who are low in self-reported executive control [17]. While the above findings are based on a small number of studies with small sample sizes, and the control groups used were of partly restricted comparability (e.g., with regard to educational level), they support RS as an important concept for understanding BPD pathology.

### History of physical and emotional abuse and neglect during childhood

A number of studies have consistently shown physical and emotional neglect and abuse during childhood and adolescence to be influential environmental factors in the development of BPD. Early evidence was provided by Linehan [22], who emphasized the relevance of an invalidating environment in her bio-social theory about the development of BPD. Early experiences of emotional neglect by primary caretakers are an important psychosocial risk factor for the development of BPD, and are one of the central features in the histories of BPD patients [23-25]. Such patients also have high rates of a history of physical abuse (around 53%), and are at high risk of developing posttraumatic stress disorder [26]. The exact nature of the relationship between maltreatment and BPD is still under debate. Bornovalova et al. [27] recently proposed that there may be a genetic influence behind the association of traumatic events and BPD, rather than BPD being directly caused by trauma.

RS has also been shown to develop in consequence of early traumatic experiences [28,29]. Horney [30] proposed that an anxiety about maltreatment develops through early rejection experiences and predisposes people to a painful sensitivity to ‘any rejection or rebuff no matter how slight’. Because childhood maltreatment constitutes experience of rejection in a very strong form [6], it may lead to RS, which in turn is associated with subsequent development of mental disorders [28]. Empirical support for this theory is given for example by Luterek et al. [31], who demonstrated the mediating role of RS on the effect of childhood sexual abuse on depressive symptoms. Based on these findings, we set out to investigate whether RS explains the relation of physical and emotional abuse and neglect during childhood and BPD symptom severity.

### Self-esteem

In DSM-5, one of the diagnostic criteria for BPD is identity disturbances, defined as a ‘markedly and persistently unstable self-image or sense of self’. An important sub-domain of self-image is the construct of self-esteem [32]. Self-esteem is low in BPD patients compared to healthy controls [33] and major depressive patients [34] and has been linked with BPD features in healthy individuals [35-37]. In general, individuals aim to maintain high self-esteem and try to defend it if it is under threat. Sociometer theory views self-esteem as an affective state that reflects whether an individual is included or is suited to be included in social groups of important others [38,39]. It describes self-esteem as a continuous online monitor of belonging, which also reports potential threat to belonging, which motivates people to keep up or regain their inclusionary status. Self-esteem is also assumed to be dependent on the evaluation of others, due to its relevance for social inclusion (for further discussion, see [40]).

Based on empirical findings, Downey and Feldman proposed a direct link between RS and self-esteem [1] and this has been confirmed in additional studies (e.g. [41,42]). Moreover, Kashdan et al. [41] propose that low self-esteem is associated with enhanced sensitivity to the valence of social feedback. These assumptions are supported by Onoda et al. [42], who found that low self-esteem influences reactions to rejection. Leary et al. [38] postulated a causal relationship between the experience of social rejection and the level of self-esteem, arguing that people who consistently tend to perceive rejection by others develop low self-esteem, whereas people who consistently tend to perceive acceptance and inclusion by others develop high self-esteem. If we conceptualize RS as an interpersonal vulnerability characterized by a physiologically based defensive motivational system [3], high RS would predispose subjects towards the perception and processing of potential rejection, and should therefore directly influence self-esteem. Based on these findings, we hypothesized that self-esteem, i.e. a sociometer giving continuous feedback regarding the inclusionary status, mediates the effects of the RS-defensive system on psychopathology.

Since low self-esteem is linked to both RS and BPD, it may account for the effect of RS on BPD symptom severity. Previous studies indicated increased rejection sensitivity as well as heightened experience of early adverse experiences and low self-esteem in BPD. However, to the best of our knowledge, no study investigated the relationship of these variables. Therefore, the aim of the present study was to gain further insight into the relationship of RS and BPD. Based on the findings described above, we hypothesized that BPD patients would report higher RS than healthy controls; that BPD symptom severity is linked to a history of childhood maltreatment and that this association is mediated by RS; and that self-esteem is closely related to both RS and BPD symptom severity and constitutes a mediator in the relationship between the two. Our study seeks to answer the following questions: 1) whether RS mediates the link between childhood abuse and neglect and BPD symptom severity; 2) whether self-esteem mediates the link between RS and BPD symptom severity; and 3) whether an increased RS is linked specifically to BPD symptomatology in the acute phase of the disorder or whether it persists into remission. Gunderson et al. [43] found that BPD is characterized by high rates of remission, however, impairment in social functioning often persists even after successful treatment of borderline symptoms (see also [44]). Since RS is defined as a cognitive affective disposition of an individual, we hypothesize that RS is still increased in BPD patients after remission and that it is linked comparably to the acutely ill patients to childhood abuse and neglect as well as self-esteem. To test this hypothesis, we included a sample of remitted BPD patients.

## Methods

### Sample

This study combined questionnaire data obtained from several ongoing studies at the Department for Psychosomatic Medicine and Psychotherapy, CIMH Mannheim. Recruitment was done through the database at the Department for Psychosomatic Medicine and Psychotherapy, CIMH Mannheim, as well as through newspaper advertisements and postings on online BPD groups. A total of 167 female subjects were enrolled, of whom 92 were outpatients and 75 were healthy controls (HC) with no lifetime or current psychiatric diagnoses. Within the patient population, 77 subjects had a diagnosis of acute BPD (at least five of the nine DSM-IV criteria: BPD-A group), while 15 had a lifetime but no current diagnosis of BPD (remitted BPD patients, less than three DSM-IV criteria in the past two years: BPD-R group). The three groups were matched for age (HC: 26.8 ± 6.6; BPD-A: 28.3 ± 6.3; BPD-R: 29.2 ± 4.7; F[1,167] =1.6, p = .206) and for years of education (HC: 12.3 ± 1.4; BPD-A: 11.9 ± 1.5; BPD-R: 11.7 ± 1.6; F[1,167] =3.7, p = .154). Data of 40 subjects have previously been included in the sample of Domsalla et al. [15].

The diagnosis of BPD according to DSM-IV was made by trained clinical psychologists using the International Personality Disorder Examination (IPDE; [45]); Axis I disorders were assessed using the Structured Interview for DSM-IV (SCID-I; [46]). A total of 16.9% of the patients in the BPD-A group and 6.7% in the BPD-R group received psychopharmacological treatment. General exclusion criteria included a lifetime history of psychotic or bipolar I disorder, current substance abuse or addiction, current pregnancy, history of organic brain disease, skull or brain damage, or severe neurological illness; and additional exclusion criteria for the BPD-R group were more than two events of non-suicidal self-injury or inpatient crisis intervention within the last two years. The co-morbidities seen in the BPD-A and BPD-R groups are shown in Table 1.

**Table 1:** **Sample characteristics – comorbidities**

	**BPD-A**		**BPD-R**	
*Current co-diagnosis*	N	%	N	%
*Major depression*	10	13,0	1	6,7
*Bipolar II*	0	0	0	0
*PTSD*	22	28,6	1	6,7
*Panic Disorder*	14	18,2	0	0
*Social Phobia*	23	29,9	2	13,3
*Specific Phobia*	13	16,9	0	0
*OCD*	8	10,4	0	0
*Bulimia*	11	14,3	0	0
*Anorexia*	4	5,2	0	0
*Substance Abuse/dependence*	0	0	0	0
*Lifetime co-diagnosis*				
*Major depression*	66	85,7	12	80,0
*Bipolar II*	3	3,9	0	0
*PTSD*	28	36,4	5	33,3
*Panic Disorder*	16	20,8	1	6,7
*Social Phobia*	31	40,3	4	2,7
*Specific Phobia*	14	18,2	1	6,7
*OCD*	8	10,4	0	0
*Bulimia*	19	24,7	4	2,7
*Anorexia*	16	20,8	3	20,0
*Substance Abuse/dependence*	33	42,9	4	2,7

### Measurements

**Rejection sensitivity** was assessed using a German version of the Rejection Sensitivity Questionnaire (RSQ) for adults [47]. The questionnaire consists of nine items that describe interpersonal scenarios in which the subject asks for help or support. The cognitive component of RS, rejection expectancy, is assessed by rating how strongly the subject expects a response of either acceptance or rejection from others. The affective component, rejection anxiety, is assessed by a question on how anxious or concerned the subject would be regarding this response. For each of the 9 scenarios, the cognitive and affective components are assessed on a 6-point Likert scale. The final RSQ score is the multiplicative composite of both subscales, based on the theory that behavior is determined by the expectancy of an outcome weighted by its relevance for an individual [6,48,49], and ranges from 1 to 36, with a high score indicating high RS. In the present study, internal consistency was high for the total score (Cronbach’s α = .87) as well as for the two subscales (rejection anxiety: α = .84, rejection expectancy α = .90). Consistency was also seen when only the data of the healthy subjects were analyzed (RSQ score: α = .75; rejection anxiety: α = .78; rejection expectancy: α = .75).

**BPD symptom severity** was assessed using the short version of the Borderline Symptom List (BSL-23; [50]; Cronbach’s α = .94), a self-rating instrument that assesses symptom severity of borderline-specific symptomatology in the last week. It contains 23 items that are rated on a 5-point Likert scale.

**Childhood abuse and neglect** were assessed using the Childhood Trauma Questionnaire (CTQ; [51]). Subjects rate the frequency of maltreatment in 28 items using a 5-point Likert scale. Items are combined to form five subscales that assess the frequency of emotional abuse (Cronbach’s α = .89), emotional neglect (Cronbach’s α = .89), physical abuse (Cronbach’s α = .82), physical neglect (Cronbach’s α = .66), and sexual abuse (Cronbach’s α = .92). Subscale scores range from 5 to 25.

**Self-esteem** was assessed using the Rosenberg Self-Esteem Scale (SES; [52]; Cronbach’s α = .84), a self-rating instrument that assesses global self-esteem. It contains ten items that are rated on a 4-point Likert scale.

**Depressive symptoms** were assessed using the Beck Depression Inventory (BDI [53], Cronbach’s α = .88), a self-report questionnaire that assesses severity of depressive symptoms in the last week. It contains 21 items comprising four statements each.

### Statistical analysis

All analyses were performed using SPSS (version 20; SPSS Inc., USA). To test whether BPD-A, BPD-R, and HC subjects differed in scores on the above questionnaires and in age, we applied one-way analyses of variance (ANOVAs). For educational level, group differences were tested using non-parametric ANOVA. Post hoc analyses were performed by pair-wise comparisons (Bonferroni-corrected for multiple testing). The overlap between the distributions of the RSQ-scores in the different groups was calculated based on the estimation of effect sizes (Cohen’s d). To determine the overlap between two groups, the area under the standard normal distribution to the right of d was checked in a z-table and then doubled [54]. To analyze the hypothesized co-variations between the clinical variables, Pearson’s correlation coefficients were computed. To assess significance between different correlations, we transformed Pearsons’s r to Fishers z-scores and tested these between groups for significance. Following the definition of a mediator given by Baron and Kenny [55] hierarchical regression analyses were computed for the mediational analyses, using the SOBEL script applicable in SPSS [56].

To determine mediational analyses, we first computed the direct effects of the predictor variable (X) on the dependent (Y) and on the potential mediator (M) variable (b(YX) and b(MX)). In a second step, we tested the influence of the mediator on the dependent variable when the predictor is considered simultaneously as predictor (b(YM.X)), as well as the influence of the predictor on the dependent variable when the mediator is considered simultaneously as predictor (b(YX.M)). A mediation can be assumed if there is an indirect effect of the predictor on the dependent variable through the mediator variable (the product of b(MX)*b(YM.X)). Preacher & Hayes [56] suggested to additionally test whether the indirect effect differed from zero. In accordance with their recommendations, we applied the SOBEL z-test using normal distribution as well as the non-parametric bootstrapping method with n = 1000 resamples. They recommend the additional use of the bootstrapping method because the SOBEL z-test is less conservative with small sample sizes and the associated risk of a violation of the normal distribution of the indirect effect (for further detail see [56]). In case of small sample size (such as in the BPD-R group), the indirect effect should only be interpreted, if statistical significance can be confirmed by the bootstrapping method.

## Results

### Comparison between groups in the RSQ

**Figure 1:**
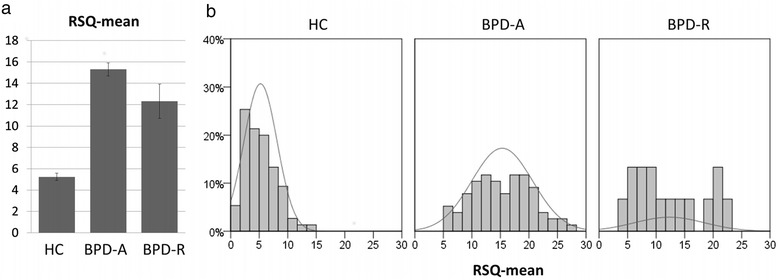
**RSQ scores for HC, BPD-A and BPD-R. a)** mean and standard error, **b)** frequency distribution.

One-way analysis of variance revealed group differences in RSQ scores (F(2,164) = 96.9, p < .001; see Table 2 and Figure 1). The scores were significantly higher for both acute and remitted BPD patients compared to HC subjects (p < .001 for both), and were higher for the BPD-A group than for the BPD-R group with a difference that approached significance (p = .060). Effect sizes indicated an overlap of RSQ score distributions of 23% for HC and BPD-A (Cohen’s d = 2.4), of 45% for HC and BPD-R (Cohen’s d = 1.5), and of 80% for BPD-A and BPD-R (Cohen’s d = 0.5).

**Table 2:** **Sample description for healthy controls (HC), acute BPD patients (BPD-A) and BPD patients in remission (BPD-R)**

	**HC**		**BPD-A**		**BPD-R**		**Anova**		**HC –BPD-A**		**HC- BPD-R**		**BPD-A-BPD-R**	
	**(N = 75)**		**(N = 77)**		**(N = 15)**									
	AM	SD	AM	SD	AM	SD	F	p	t	p	t	p	t	p
RSQ	5.2	±2.9	15.3	±5.3	12.3	±6.2	96.9	<.001	14.42	<.001	6.91	<.001	1.94	.056
*RSQ-A*	2.2	±0.6	4.1	±0.9	3.6	±1.3	108.4	<.001	15.67	<.001	4.21	.001	1.62	.124
*RSQ-E*	2.0	±0.7	3.4	±0.8	3.0	±1.0	60.5	<.001	−11.22	<.001	−3.93	.001	−1.38	.172
BSL	0.1	±0.2	1.9	±0.7	0.8	±0.7	194.4	<.001	20.61	<.001	3.69	.003	5.00	<.001
BDI	2.5	±2.9	23.1	±10.3	11.0	±8.0	136.2	<.001	16.65	<.001	3.97	.001	4.17	<.001
Self-Esteem	26.7	±4.1	10.1	±8.6	15.9	±9.0	107.7	<.001	−15.18	<.001	−4.51	<.001	−2.38	.019
CTQ-total	30.7	±7.2	61.9	±17.7	61.3	±18.7	97.2	<.001	13.24	<.001	5.83	<.001	0.11	.915
*CTQ-physical abuse*	5.6	±1.7	8.5	±4.5	6.9	±4.1	13.3	<.001	5.17	<.001	1.17	.263	1.19	.236
*CTQ-physical neglect*	5.8	±1.6	10.0	±3.7	11.1	±3.6	44.5	<.001	8.75	<.001	5.19	<.001	−0.96	.338
*CTQ-emotional abuse*	6.7	±2.5	16.9	±5.1	16.0	±5.7	119.2	<.001	15.49	<.001	5.80	<.001	0.60	.551
*CTQ-emotional neglect*	7.4	±3.2	17.1	±4.8	17.3	±6.5	101.4	<.001	14.21	<.001	5.43	<.001	−0.12	.901
*CTQ-sexual abuse*	5.3	±1.3	9.0	±5.1	10.0	±5.7	20.4	<.001	5.96	<.001	2.98	.011	-.63	.530

To assess whether group differences in RSQ scores could be completely explained by BPD symptom severity, we conducted the same analysis with BSL scores as a covariate (Table 3). The results of this analysis were comparable (F(2,164) = 76.11, p < .001), with significantly higher RSQ scores in both BPD groups compared to HC (p < .001 for all). Here, however, no differences were seen between acute and remitted BPD patients after controlling for borderline symptom severity (p > .999).

**Table 3:** **Pearson’s correlation coefficients between Rejection Sensitivity (RSQ), borderline symptom severity (BSL-23), childhood maltreatment (CTQ), self-esteem (SES) and depressive symptoms (BDI) for healthy controls (HC), acute BPD patients (BPD-A) and BPD patients in remission (BPD-R)**

	**BSL**			**CTQ**			**SES**			**BDI**		
	**HC**	**BPD**		**HC**	**BPD**		**HC**	**BPD**		**HC**	**BPD**	
		**-A**	**-R**		**-A**	**-R**		**-A**	**-R**		**-A**	**-R**
RSQ	.24*	.30**	.62*	.55***	.20	.33	-.41***	-.45***	-.77**	.30**	.20	.78**
BSL	-	-	-	.27*	.04	.25	-.37**	-.52***	-.81***	.53***	.77***	.68**
CTQ				-	-	-	-.24*	-.19	-.41	.30*	-.06	.20
SES							-	-	-	-.47***	-.47***	-.83***

*=p<.05, **=p<.01, ***=p<.001.

An explorative comparison of group differences in the components of rejection expectancy (RS-E) and rejection anxiety (RS-A) revealed a similar pattern. Both RS-E and RS-A were increased in the two patient groups compared to the HC group (ANOVA: RS-E: F(2,164) = 60.5, p < 0.001; RS-A: F(2,164) = 108.4,, p < .001; p < .001 for all post hoc tests). Effect sizes suggest that the highest differentiation between BPD-A and HC is in rejection anxiety (d = 2.54, RS-E: d = 1.82, HC compared to BPD-R RS-A: d = 1.5, RS-E: d = 1.24). Post hoc analyses revealed a trend for higher RS-A in acute as compared to remitted BPD patients (p = .057, Cohen’s d = 0.51) and a statistically non-significant difference in RS-E (p = 0.397, Cohen’s d = 0.37). Analogous to the total RS score analysis, differences between patient groups disappeared after controlling for borderline symptom severity (p > .999).

RS-E and RS-A were highly correlated in the total sample, with Pearson’s r = .78 (p < 0.001). Correlation analyses for the three groups separately revealed significant correlations for all groups (HC: r = 0.70, p < 0.001; BPD-A: r = 0.56, p < 0.001; BPD-R: r = .716, p = .004).

### Covariation of RSQ with borderline symptom severity

**Figure 2:**
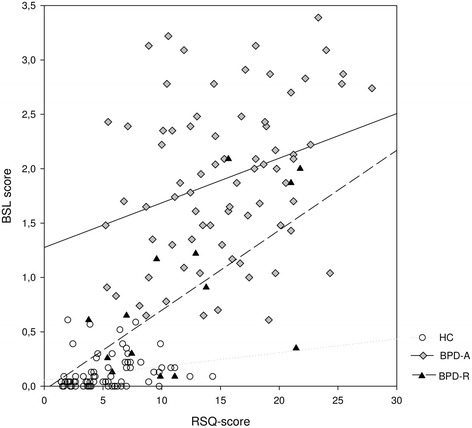
**Correlation between RSQ and Borderline symptom severity assessed by the BSL together with regression lines for HC, BPD-A and BPD-R.**

Rejection sensitivity correlated with BPD symptom severity as assessed with the BSL in all three groups (Table 3 and Figure 2). The strength of the correlation did not differ significantly between groups (p > .1 for all).

### RSQ, childhood maltreatment, and borderline symptom severity

In the HC group, higher scores on the CTQ were linked with higher scores on the BSL, the RSQ, and the BDI (see Table 3). These associations were not seen in either of the BPD groups.

For explorative purposes, we additionally calculated separate correlations for each subscale of the CTQ. In the BPD groups, only a positive correlation of the RSQ and CTQ-‘physical neglect’ in the BPD-A group could be observed (r = .27, p < .05). RSQ did not correlate with any of the other CTQ subscales (p > .1 for all). Because of this lack in co-variation, no mediation analysis was calculated for the two BPD groups.

In contrast, in the HC group, higher RSQ scores were linked to higher CTQ scores in all CTQ subscales, with the highest correlation seen with the subscale ‘emotional neglect’. Similarly, BSL scores increased with CTQ scores. To further specify the associations between childhood maltreatment, borderline symptomatology, and RS in the HCs, we calculated a hierarchical regression. However, neither the SOBEL-z-test nor the non-parametric bootstrapping method revealed a mediating effect of the RSQ in the association of the CTQ and the BSL (z = .93; p = .353).

### RSQ, self-esteem and borderline symptom severity

RSQ, SES, and BSL were closely related in all subgroups of our sample (see Table 3). To test whether the association between RS and borderline symptom severity is mediated by self-esteem, we applied a hierarchical regression with a subsequent SOBEL-z-test and a non-parametric bootstrapping method.

**Figure 3:**
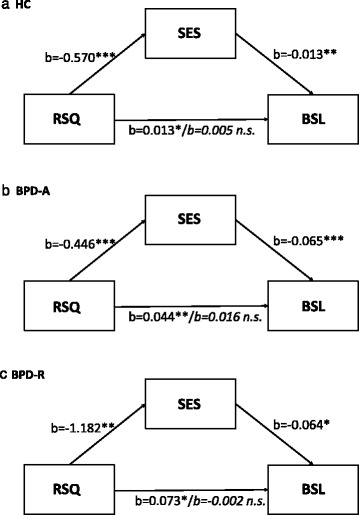
**Results of the hierarchical regression analysis with rejection sensitivity (RSQ), BPD symptom severity (BSL) and self-esteem (SES) for healthy controls (a.; HC) and acute (b.; BPD-A) and remitted (c.; BPD-R) BPD patients.**

The hierarchical regression comprised three steps: 1) the BSL score was predicted by the RSQ, 2) self-esteem was predicted by the RSQ, and 3) the BSL score was predicted simultaneously by the RSQ and the SES. Results were similar for all three groups. The RSQ score was a significant predictor for both the BSL score (BPD-A: b = .04, t = 0.89, p = .005; BPD-R: b = .07, t = 2.76, p = .017; HC: b = .01, t = 2.09, p = .039) and the SES score (BPD-A: b = −.47, t = −4.20, p < .001; BPD-R: b = −1.18, t = −4.29, p = .001; HC: b = −0.57, −3.98, p < .001). When predicting the BSL score simultaneously by the RSQ and the SES, only the SES remained a significant predictor (BPD-A: b = −.06, t = −4.18, p < .001; BPD-R: b = −.06, t = −2.9, p = .015; HC: b = −.01, t = −2.65, p = .010), whereas the effect of the RSQ diminished (BPD-A: b = .02, t = 1.01, p = .318; BPD-R: b = −.002, t = .05, p = .959; HC: b = .01, t = .84, p = .404). The SOBEL z-test revealed an indirect effect of RSQ on BSL through SES in all groups that was significantly higher than zero (BPD-A: z = 2.12, p = .004; BPD-R: z = 2.36, p = .018, HC: z = 2.16, p = .031). See Figure 3.

To account for violations of the normal distribution of rating scores in the HC and BPD-A groups, a non-parametric bootstrapping method was additionally used to estimate the confidence interval of the indirect effect. The indirect effect was different from zero with a probability of 99% for both patient groups. For the HC, the bootstrapping method could not confirm the mediating effect revealed by the SOBEL z-test.

To control for the effects of depressive symptoms on the association between RSQ and BSL, we calculated mediational analyses using the BDI scores as mediators. In both patient groups, neither the SOBEL z-test nor the bootstrapping method supported the existence of an indirect effect of RSQ on BSL through the BDI (p > .05). In contrast, in the HC group, the SOBEL z-test indicated that the BDI score mediates the effect of RSQ on BSL (z = 2.25, p = .025), although this could not be confirmed by the bootstrapping method. To take into account the influence of depressive symptoms on the mediating role of self-esteem in the relation between RS and borderline symptom severity, we repeated the main analyses with self-esteem scores corrected for depressive symptoms. Since borderline and depressive symptoms share a large portion of variance due to an overlap of symptoms [57], we corrected BDI scores for common variance with BSL scores, and used the resulting BDI residuals in a second step to correct self-esteem scores for depressive symptoms. Mediational analysis using these corrected self-esteem scores confirmed our findings: the SOBEL z-test revealed an indirect effect of RSQ on BSL through the SES in all three samples. In both patient groups, but not in the HC group, this finding was confirmed using the bootstrapping method.

## Discussion

This study aimed to investigate rejection sensitivity in acute and remitted BPD patients and the relation of this cognitive affective disposition with childhood maltreatment and self-esteem.

As we hypothesized, BPD patients reported markedly higher RS compared to healthy individuals. Both increased rejection expectancy and increased rejection anxiety contributed to this effect; i.e., BPD patients had a higher expectation of being rejected, and were more anxious about this expectation. These findings agree with previous studies that indicated a low overlap of RSQ scores in BPD and healthy subjects and thus a high separation of these groups [13-15].

We included BPD patients in both acute and remitted stages of the disorder in order to explore whether high RS is a stable feature of BPD. Our data revealed that an enhanced RS persists in BPD after remission from acute psychopathological symptoms. BPD subjects in remission tended to show lower RS compared to the acute BPD group, but still reported higher RS compared to the HC group.

In general, RS co-varied with BPD symptom severity, suggesting that this disposition is modulated by the actual psychopathological state. To explore whether the trend towards reduced RS in the group of remitted patients can be explained by the lower severity of BPD symptoms, we compared RSQ scores between groups using BSL scores as a covariate. After this correction, BPD patients still scored higher in RS than healthy individuals, but RS was not distinguishable between acute and remitted BPD patients. This suggests that the trend towards an attenuation of RS during remission is related to a reduction of BPD symptoms in these patients, which may to be expected due to the conceptual overlap of BPD symptoms and rejection sensitivity. However, beyond the influence of current psychopathological symptoms, BPD patients had an increased RS as a stable feature which was not solely explainable by an overlap with current symptom severity of BPD psychopathology.

Our findings confirm that RS is linked not only to the number of BPD features, as has been shown in earlier studies [17-21], but that it is also related to BPD symptom severity in a clinical sample of BPD patients. Current BPD symptoms accentuate the severity of RS, which can be assumed to be trait-like increased in these patients. To gain further insight into trait and state-related portions of RS, longitudinal studies are required that investigate variations in RS over time, as well as its sensitivity to change after therapeutic interventions, and its dependency from modulating factors such as social and non-social stress [58].

Further, we addressed the question of whether BPD symptom severity is linked to a history of childhood maltreatment and whether this association is mediated by RS. Here, our findings contradicted our hypothesis. Both acutely ill and remitted BPD patients reported higher frequencies of childhood maltreatment compared to the healthy subjects. However, in the clinical samples, we found no co-variation between either the CTQ and BPD symptom severity or between CTQ and RS. By contrast, an association between CTQ and RS was seen for the healthy individuals. The fact that the HC group showed the highest correlation for the RSQ and the CTQ for the subscale emotional neglect, suggests that emotional neglect indeed seems to more profoundly undermine development compared to other types of childhood maltreatment [59]. Chamberland et al. [60] showed that emotional neglect even is a stronger predictor of problems such as lack of self-esteem and interpersonal difficulties compared to other forms of maltreatment (e.g. physical abuse, sexual abuse).

One reason for the missing relation between CTQ, BPD symptom severity, and RS in our clinical sample as well as the divergent findings between HCs and the clinical groups may be that the level and variability of measurement score differ between groups. Our findings may have been caused by a restricted data range in the BPD groups. However, since ranges in all variables are lower in the HC group than in both BPD groups, differences in variability of measurements do not account for our findings. Nevertheless, one might speculate whether these findings can be explained by a non-linear relation between the frequency of trauma experiences during childhood and the severity of psychopathological symptoms and RS. Experiences of maltreatment within a low range seem to affect psychopathology and RS in healthy subjects as well as in clinical samples such as patients suffering from depression [28,29,31]. If a threshold is reached, however, there may be a qualitative switch into BPD, possibly linked to specific neurobiological or environmental factors that increase an individual’s vulnerability for BPD. Recent studies suggest that interactions between childhood emotional abuse and personality traits such as sociability and neuroticism as well as emotion regulation difficulties have to be considered in order to explain differences in the symptom severity in BPD patients [61-63]. Further studies are required that address the relation between childhood maltreatment and BPD symptom severity in clinical samples that take these potentially modulating features into account. Beyond that, a more elaborate method such as the MACE-interview (Maltreatment and Abuse Chronology of Exposure scale developed by Teicher and Parigger [64]) compared to the self-report based CTQ may be essential to measure differences in frequency and severity of childhood maltreatment. This would allow researchers to better differentiate individuals with a history of childhood maltreatment before looking at possible associations with symptom severity, rejection sensitivity or self-esteem.

Finally, we were interested in the relation between self-esteem, RS, and BPD symptom severity, as well as the possible role of self-esteem as a mediator in the relation between RS and BPD symptom severity. In general, self-esteem was reduced in both acutely ill and remitted BPD patients compared to the healthy individuals and lower self-esteem was reported in acute compared to remitted BPD patients. Consistent with our hypothesis, reduced self-esteem was found to be linked to both increased BPD symptom severity and higher RS and mediated the relation between both and this held true for both clinical samples. According to the sociometer theory, self-esteem constitutes a monitoring system that signals whether an individual is accepted or rejected by others to enhance the probability of survival [39]. It is assumed that these experiences are accumulated over time to form trait self-esteem. Chronic feelings of low or high self-esteem influence the development of beliefs and social motivations, which in turn modulate trait self-esteem [48]. These factors might be responsible for the mediating role of self-esteem in the relation of RS and BPD symptom severity. If future studies can support this interpretation and identify the relevant beliefs, these might constitute an essential aspect that has to be considered when trying to influence the disadvantageous effect of RS during therapeutic interventions.

An example for a specific motivation is the defense of rejection. Individuals low in self-esteem use their knowledge about the security of acceptance to guide their social behavior [48,65]. This interaction between high RS and low self-esteem might result in the social relationships typically seen in BPD patients, who have less frequent social contacts, and often most of these contacts are with other BPD patients or with the therapist (see also [66]). To overcome this pattern, patients would need more security of acceptance to participate in social interactions, but the enhanced RS prohibits exactly this feeling of security about being accepted by others. Successful treatment should try to disrupt this vicious circle of enhanced RS and low self-esteem.

Park et al. have presented first evidence that there are indeed strategies to attenuate the negative effects of threat on people high in RS [67]. They investigated appearance-based RS, which constitutes a subtype of RS and is defined as anxious concerns and expectations about being rejected based on ones’ physical attractiveness [67]. In the light of Anthony et al. [48], who found that traits such as physical attractiveness and popularity are particularly influential in shaping self-esteem since they generally evoke acceptance by others, further studies are required that take a closer look at appearance-based RS in BPD. In BPD patients, increasing BPD-symptomatology is linked to a stronger believe that attractiveness is relevant for acceptance [68]. BPD is also associated with a negative body image that cannot be explained by comorbid eating disorder [68-70]. The importance of the negative body image in BPD and its potential association to self-esteem and expectations regarding acceptance may constitute a promising avenue to influence RS in therapeutic interventions.

Several limitations of the present study have to be mentioned. First, the sample size of the remitted BPD patients’ group was quite small. However, since we could confirm statistically increased rejection sensitivity in the remitted patients (despite the small sample size and low statistical power), it can be assumed that the population effect is strong [71]. Nonetheless, a replication in a larger group of remitted patients seems necessary to further explore whether our findings may depend on specific features of the current sample and how co-morbidities may influence RS during remission. Especially repeated measurements of RS over the course of illness seem essential to deepen our understanding of the trait or state-like nature of RS in BPD. Gunderson et al. [43] found that impairment in social functioning often persists even after successful treatment of borderline symptoms (see also [44]). Studies that address the underlying alterations in social cognitive functioning seem to be essential to further improve therapeutic interventions. However, the investigation of BPD patients in remission has been widely neglected so far. Our findings emphasize the need for studies in this subsample of BPD patients and suggest that rejection sensitivity may contribute to persisting social dysfunction in that it may bias social encounters by anxious expectations of rejection after improvement of acute BPD symptoms.

Besides, the highly heterogeneous nature of the investigated BPD samples with regard to co-morbidities may have clouded our results [72-74]. To test the specificity of our findings, further studies including clinical control groups are required. To assess the effect of coexisting PTSD and social anxiety disorder, we calculated additional analyses which are reported in the supplementary material (see Additional file 1: Tables S1-6). Although these analyses suggest that our findings cannot be attributed to co-diagnoses of PTSD, comorbidity with social anxiety disorder was nevertheless essential for the relation between RS and BPD symptom severity. Interestingly, anxiety disorders and especially social anxiety disorder are frequent comorbidities in BPD and such a comorbidity can better discriminate between BPD and patients with other personality disorders than e.g. a comorbid mood disorder [73]. Further studies should aim at even larger sample sizes to allow for the analysis of complex interactions between different comorbidities and psychopharmacological treatment with a special focus on the relevance of a coexisting social anxiety disorder. Using a group of patients with social anxiety disorder without a comorbid BPD as a clinical control group also is important.

Additionally, the cross-sectional nature of the present study has to be emphasized. Despite its usefulness for gaining first insight into important interrelations between constructs relevant for understanding BPD, it seems worth noting that the variables in question are assessed at only one point in time. Since no reliable causal inferences can be derived from this, a longitudinal design would be desirable for the further investigation of the relationship between RS, self-esteem and BPD.

On the other hand, our findings have strong implications for the development of intervention strategies in BPD. While improvement of self-esteem is already a goal in psychotherapeutic approaches such as dialectical behavioral therapy [22], RS has not been directly addressed so far. Our data suggest that an intervention should target both the cognitive and the affective component of RS. Since self-esteem mediates the relation between RS and the severity of BPD symptoms, every intervention that aims towards improving RS has to integrate an improvement of self-esteem to achieve a beneficial effect.

Another important aspect may be that RS influences learning about social threat in healthy subjects. Olsson et al. [75] demonstrated that high RS is linked to diminished extinction of previously conditioned fear responses, which is specific for social stimuli of negative valence. They propose that this mechanism prevents an updating of acquired expectations for threat. Although no data are yet available that demonstrates a comparable effect in BPD, one may assume that RS operates in BPD in a comparable manner, resulting in a vicious circle of self-fulfilling prophecies.

It must be mentioned that both the literature review and the findings of the present study rely solely on questionnaire data; i.e., self-reports that reflect the subjective expectancy of rejection in social encounters. While RS is a concept that emphasizes subjective expectancies and emotions, it still must be clarified whether the increased RS in BPD reflects primarily a biased view or whether it adequately describes the true behavior of the respondent’s interaction partners. A differentiation between these alternatives seems necessary for the design of specific therapeutic interventions. In case of a biased perspective of social encounters, interventions will have to involve a cognitive restructuring of expectancies, accompanied by training for the perception of positive cues. In case of a realistic evaluation of the behavior of others, coping strategies with social rejection and the analyses of disadvantageous social actions should constitute the focus, in order to help the patient alter the course of his or her interactions with others.

Both the above explanations may contribute to high RS in BPD. In healthy individuals, studies on RS support the idea of a biased perspective of individuals high in RS: adolescents with high RS overestimated rejection and felt more victimized by their peers, despite not being seen by their peers as being more victimized [76]. Data from experimental studies on the experience of social encounters in BPD point in a similar direction: BPD patients felt more excluded in social situations during which they had in fact been included, or during which the behavior of the social partners was determined by predefined rules and not by the partners’ intentions [33,56,77]. However, other studies suggest that social rejection by others may indeed occur more frequently with BPD patients.

The increased frequency of being rejected is already part of the RS model by Levy et al. [78]. Based on a hypersensitivity to possible rejection, cognitive and emotional reactions may result in maladaptive behavior such as hostility and withdrawal, which might in turn provoke actual rejection by others. A review by Sansone and Sansone [79] suggests that negative perceptions of and emotional responses toward BPD patients are common in mental health clinicians, and that these may ‘simply reflect a very human reaction to the complex and pathological behaviors of these patients’. So far, no data are available that directly link RS to the interpersonal deficits in BPD. Future studies will have to address this topic by investigating, for example, the link between RS and problems in intimate relationships as it has been demonstrated to exist in non-clinical samples [1].

## Conclusions

In sum, RS seems to be a promising concept to add to our understanding of interpersonal functioning in both acute and remitted BPD patients. However, further studies are required to extend our understanding of the relevance of RS for BPD psychopathology. Downey et al. [1] emphasized in their revised RS model that rejection expectancy may be linked not only to anxiety but also to anger. Both emotions have been linked to different behavioral responses; i.e., withdrawal or reactive aggression, and the intensity of these responses is differentially affected by the nature of the rejection situation. One example is the degree of ambiguity of the rejection situation. In situations of ambiguous rejection, individuals with high rejection expectancies responded with accentuated reactive aggression, while in situations of overt rejection, individuals with high rejection anxiety showed the strongest withdrawal [80]. Future studies will have to test the relevance of the complex interplay of cognitive, emotional, and behavioral components of rejection sensitivity and their consequences for the understanding of interaction behaviors in this patient group.

## Additional file

Additional file 1:
**Subgroup analyses.**

## References

[1] Downey G, Feldman SI (1996). Implications of rejection sensitivity for intimate relationships. J Pers Soc Psychol.

[2] Downey G, Mougios V, Ayduk O, London BE, Shoda Y (2004). Rejection sensitivity and the defensive motivational system: insights from the startle response to rejection cues. Psychol Sci.

[3] Pietrzak J, Downey G, Ayduk O, Baldwin MW (2005). Rejection sensitivity as an interpersonal vulnerability. Interpersonal cognition.

[4] Cacioppo JT, Gardner WL (1999). Emotion. Annu Rev Psychol.

[5] Lang PJ, Bradley MM, Cuthbert BN (1990). Emotion, attention, and the startle reflex. Psychol Rev.

[6] Downey G, Feldman S, Khuri J, Friedman S, Reynolds WM, Johnston HF (1994). Maltreatment and childhood depression. Handbook of depression in children and adolescents.

[7] London B, Downey G, Bonica C, Paltin I (2007). Social causes and consequences of rejection sensitivity. J Res Adolescence.

[8] Butler JC, Doherty MS, Potter RM (2007). Social antecedents and consequences of interpersonal rejection sensitivity. Personal Individ Differ.

[9] Brendgen M, Vitaro F, Tremblay RE, Wanner B (2002). Parent and peer effects on delinquency-related violence and dating violence: a test of two mediational models. Soc Dev.

[10] McLachlan J, Zimmer-Gembeck MJ, McGregor L (2012). Rejection sensitivity in childhood and early adolescence: peer rejection and protective effects of parents and friends. Journal of Relationships Research.

[11] Romero-Canyas R, Downey G (2013). What I see when I think it’s about me: people low in rejection-sensitivity downplay cues of rejection in self-relevant interpersonal situations. Emotion.

[12] Rosenbach C, Renneberg B (2011). Rejection, excluded, ignored: the perception of social rejection and mental disorders - a review. Verhaltenstherapie.

[13] Staebler K, Helbing E, Rosenbach C, Renneberg B (2010). Rejection sensitivity and borderline personality disorder. Clin Psychol Psychother.

[14] Berenson KR, Downey G, Rafaeli E, Coifman KG, Paquin NL (2011). The rejection-rage contingency in borderline personality disorder. J Abnorm Psychol.

[15] Domsalla M, Koppe G, Niedtfeld I, Vollstadt-Klein S, Schmahl C, Bohus M, et al. Cerebral processing of social rejection in patients with borderline personality disorder. Social cognitive and affective neuroscience*.* 2013, epub ahead of print. 10.1093/scan/nst176 PMC4221221 24273076

[16] Miano A, Fertuck EA, Arntz A, Stanley B (2013). Rejection sensitivity is a mediator between borderline personality disorder features and facial trust appraisal. J Pers Disord.

[17] Ayduk O, Zayas V, Downey G, Cole AB, Shoda Y, Mischel W (2008). Rejection sensitivity and executive control: joint predictors of borderline personality features. J Res Pers.

[18] Boldero JM, Hulbert CA, Bloom L, Cooper J, Gilbert F, Mooney JL (2009). Rejection sensitivity and negative self-beliefs as mediators of associations between the number of borderline personality disorder features and self-reported adult attachment. Personal Ment Health.

[19] Gardner KJ, Qualter P, Stylianou M, Robinson AJ (2010). Facial affect recognition in non-clinical adults with borderline personality features: the role of effortful control and rejection sensitivity. Personal Individ Differ.

[20] Selby EA, Ward AC, Joiner TE (2010). Dysregulated eating behaviors in borderline personality disorder: are rejection sensitivity and emotion dysregulation linking mechanisms?. Int J Eat Disord.

[21] Meyer B, Ajchenbrenner M, Bowles DP (2005). Sensory sensitivity, attachment experiences, and rejection responses among adults with borderline and avoidant features. J Pers Disord.

[22] Linehan MM (1993). Cognitive-behavioral treatment of borderline personality disorder.

[23] Zanarini MC, Williams AA, Lewis RE, Reich RB, Vera SC, Marino MF (1997). Reported pathological childhood experiences associated with the development of borderline personality disorder. Am J Psychiatr.

[24] Battle CL, Shea MT, Johnson DM, Yen S, Zlotnick C, Zanarini MC (2004). Childhood maltreatment associated with adult personality disorders: findings from the Collaborative Longitudinal Personality Disorders Study. J Pers Disord.

[25] Ball JS, Links PS (2009). Borderline personality disorder and childhood trauma: evidence for a causal relationship. Curr Psychiatry Rep.

[26] Golier JA, Yehuda R, Bierer LM, Mitropoulou V, New AS, Schmeidler J (2003). The relationship of borderline personality disorder to posttraumatic stress disorder and traumatic events. Am J Psychiatry.

[27] Bornovalova MA, Huibregtse BM, Hicks BM, Keyes M, McGue M, Iacono W (2013). Tests of a direct effect of childhood abuse on adult borderline personality disorder traits: a longitudinal discordant twin design. J Abnorm Psychol.

[28] Downey G, Khouri H, Feldman S, Cicchetti D, Toth S (1997). Early interpersonal trauma and adult adjustment: the mediational role of rejection sensitivity. Rochester symposium on developmental psychopathology, volume VIII: the effects of trauma on the developmental process.

[29] Feldman S, Downey G (1994). Rejection sensitivity as a mediator of the impact of childhood exposure to family violence on adult attachment behavior. Dev Psychopathol.

[30] Horney K (1937). The neurotic personality of our time.

[31] Luterek JA, Harb GC, Heimberg RG, Marx BP (2004). Interpersonal rejection sensitivity in childhood sexual abuse survivors: mediator of depressive symptoms and anger suppression. J Interpers Violence.

[32] James W (1890). The principles of psychology.

[33] Kanter JW (2001). Finding the self: a behavioral measure and its clinical implications. Psychotherapy.

[34] Abela JR, Payne AV, Moussaly N (2003). Cognitive vulnerability to depression in individuals with borderline personality disorder. J Pers Disord.

[35] Watson DC, Sinha BK (1998). Comorbidity of DSM-IV personality disorders in a nonclinical sample. J Clin Psychol.

[36] Tolpin LH, Gunthert KC, Cohen LH, O’Neill SC (2004). Borderline personality features and instability of daily negative affect and self-esteem. J Pers.

[37] Zeigler-Hill V, Abraham J (2006). Borderline personality features: instability of self-esteem and affect. J Soc Clin Psychol.

[38] Leary MR, Tambor ES, Terdal SK, Downs DL (1995). Self-esteem as an interpersonal monitor: the sociometer hypothesis. J Pers Soc Psychol.

[39] Leary MR, Baumeister RF, Zanna MP (2000). The nature and function of self-esteem: sociometer theory. Advances in experimental social psychology.

[40] Pyszczynski T, Greenberg J, Solomon S, Arndt J, Schimel J (2004). Why do people need self-esteem? A theoretical and empirical review. Psychol Bull.

[41] Kashdan TB, Dewall CN, Masten CL, Pond RS, Powell C, Combs D (2014). Who is most vulnerable to social rejection? The toxic combination of low self-esteem and lack of negative emotion differentiation on neural responses to rejection. PLoS One.

[42] Onoda K, Okamoto Y, Nakashima K, Nittono H, Yoshimura S, Yamawaki S (2010). Does low self-esteem enhance social pain? The relationship between trait self-esteem and anterior cingulate cortex activation induced by ostracism. Soc Cogn Affect Neurosci.

[43] Gunderson JG, Stout RL, McGlashan TH, Shea MT, Morey LC, Grilo CM (2011). Ten-year course of boderline personality disorder. Arch Gen Psychiatry.

[44] Lis S, Bohus M (2013). Social interaction in borderline personality disorder. Curr Psychiatry Rep.

[45] Loranger AW (1999). International Personality Disorder Examination (IPDE): DSM-IV and ICD-10 modules.

[46] First MB, Spitzer RL, Gibbon M, Williams JBW, Benjamin LS (1997). User’s guide for the structured clinical interview for DSM-IV Axis I disorders (SCID-I) - clinical version.

[47] Berenson KR, Gyurak A, Ayduk O, Downey G, Garner MJ, Mogg K (2009). Rejection sensitivity and disruption of attention by social threat cues. J Res Pers.

[48] Anthony DB, Holmes JG, Wood JV (2007). Social acceptance and self-esteem: tuning the sociometer to interpersonal value. J Pers Soc Psychol.

[49] Rotter JB (1954). Social learning and clinical psychology.

[50] Bohus M, Kleindienst N, Limberger MF, Stieglitz RD, Domsalla M, Chapman AL (2009). The short version of the Borderline Symptom List (BSL-23): development and initial data on psychometric properties. Psychopathology.

[51] Bernstein DPFL (1998). Childhood trauma questionnaire: a retrospective self-report.

[52] Rosenberg M (1965). Society and the adolescent self-image.

[53] Beck AT, Ward CH, Mendelson M, Mock H, Erbaugh J (1961). An inventory for measuring depression. Arch Gen Psychiatry.

[54] Bortz J, Döring N (2006). Forschungsmethoden und Evaluation für Human- und Sozialwissenschaftler: mit … 87 Tabellen. 4, überarb. Aufl. edn.

[55] Baron RM, Kenny DA (1986). The moderator-mediator variable distinction in social psychological research: conceptual, strategic, and statistical considerations. JPSP.

[56] Preacher KJ, Hayes AF (2004). SPSS and SAS procedures for estimating indirect effects in simple mediation models. Behav Res Methods Instrum Comput.

[57] Paris J (2010). Effectiveness of different psychotherapy approaches in the treatment of borderline personality disorder. Curr Psychiatry Rep.

[58] Liu RT, Kraines MA, Massing-Schaffer M, Alloy LB (2014). Rejection sensitivity and depression: mediation by stress generation. Psychiatry.

[59] Egeland B, Sroufe LA, Erickson M (1983). The developmental consequence of different patterns of maltreatment. Child Abuse Neglect.

[60] Chamberland C, Fallon B, Black T, Trocmé N, Chabot M (2012). Correlates of substantiated emotional maltreatment in the second canadian incidence study. J Fam Viol.

[61] Martin-Blanco A, Soler J, Villalta L, Feliu-Soler A, Elices M, Perez V (2014). Exploring the interaction between childhood maltreatment and temperamental traits on the severity of borderline personality disorder. Compr Psychiatry.

[62] Laporte L, Paris J, Guttman H, Russell J (2011). Psychopathology, childhood trauma, and personality traits in patients with borderline personality disorder and their sisters. J Pers Disord.

[63] Carvalho Fernando S, Beblo T, Schlosser N, Terfehr K, Otte C, Lowe B (2013). The impact of self-reported childhood trauma on emotion regulation in borderline personality disorder and major depression. J Trauma Dissociation.

[64] Pechtel P, Lyons-Ruth K, Anderson CM, Teicher MH (2014). Sensitive periods of amygdala development: the role of maltreatment in preadolescence. Neuroimage.

[65] Anthony DB, Wood JV, Holmes JG (2007). Testing sociometer theory: self-esteem and the importance of acceptance for social decision-making. J Exp Soc Psychol.

[66] Clifton A, Pilkonis PA, McCarty C (2007). Social networks in borderline personality disorder. J Pers Disord.

[67] Park LE (2007). Appearance-based rejection sensitivity: implications for mental and physical Health, affect, and motivation. Pers Soc Psychol B.

[68] Sansone RA, Chu JW, Wiederman MW (2010). Body image and borderline personality disorder among psychiatric inpatients. Compr Psychiatry.

[69] Dyer A, Borgmann E, Feldmann REJ, Kleindienst N, Priebe K, Bohus M (2013). Body image disturbance in patients with borderline personality disorder: Impact of eating disorders and perceived childhood sexual abuse. Body Image.

[70] Haaf B, Pohl U, Deusinger IM, Bohus M (2001). Examination of body concept on female patients with borderline personality disorder. Psychother Psych Med.

[71] Bakan D (1966). The test of significance in psychological research. Psychol Bull.

[72] Grant BF, Chou SP, Goldstein RB, Huang B, Stinson FS, Saha TD (2008). Prevalence, correlates, disability, and comorbidity of DSM-IV borderline personality disorder: results from the Wave 2 National Epidemiologic Survey on Alcohol and Related Conditions. J Clin Psychiatry.

[73] Zanarini MC, Frankenburg FR, Dubo ED, Sickel AE, Trikha A, Levin A (1998). Axis I comorbidity of borderline personality disorder. Am J Psychiatry.

[74] Zanarini MC, Frankenburg FR, Hennen J, Reich DB, Silk KR (2004). Axis I comorbidity in patients with borderline personality disorder: 6-year follow-up and prediction of time to remission. Am J Psychiatry.

[75] Olsson A, Carmona S, Downey G, Bolger N, Ochsner KN (2013). Learning biases underlying individual differences in sensitivity to social rejection. Emotion.

[76] Zimmer-Gembeck MJ, Nesdale D, McGregor L, Mastro S, Goodwin B, Downey G (2013). Comparing reports of peer rejection: associations with rejection sensitivity, victimization, aggression, and friendship. J Adolescence.

[77] Lammers C-H, Röpke S, Dulz B (2007). Selbstwert und Borderline-Persönlichkeitsstörung. Persönlichkeitsstörungen: Theorie und Therapie.

[78] Levy SR, Ayduk O, Downey G, Leary MR (2001). The role of rejection sensitivity in people’s relationships with significant others and valued social groups. Interpersonal rejection.

[79] Sansone RA, Sansone LA (2013). Responses to mental health clinicians to patients with borderline personality disorder. Innov Clin Neurosci.

[80] Zimmer-Gembeck MJ, Nesdale D (2012). Anxious and angry rejection sensitivity, social withdrawal, and retribution in high and low ambiguous situations. J Pers.

