# Supplementary material for: Rejection sensitivity and symptom severity in patients with borderline personality disorder: effects of childhood maltreatment and self-esteem
Source: Borderline Personal Disord Emot Dysregul. 2015 Mar 20;2:4. doi: 10.1186/s40479-015-0025-x (PMC4579499; doi:10.1186/s40479-015-0025-x)
Supplement: Additional file 1: — Subgroup analyses. [file 40479_2015_25_MOESM1_ESM.pdf]

Supplementary Material

1. Analyses of the effects of comorbidities

To gain further insight into the reported effects, we run analyses on subgroups of the acute BPD sample with PTSD or social anxiety as co-morbidities.

In sum, the correlation between RS and borderline symptom severity only revealed significant within BPD patients without a current PTSD and within BPD individuals with an acute social anxiety disorder. With regard to our sample, PTSD didn't seem to impact the transmitting effect of self-esteem on the relationship between RS and BSL, as we found an indirect effect of the RSQ on the BSL through the SES score only in the BPD-PTSD– - group. BPD subjects with and without PTSD were comparable in all clinical variables. Moreover, a comorbid social anxiety disorder seems to have an influence on the relationship between RS and borderline symptom severity, as only Borderline patients with a present social anxiety disorder showed a significant association between the latter mentioned constructs, whereas those without an acute social anxiety disorder failed to show one.

These analyses have to be interpreted with caution since low power may have prevented the detection of significant relations. Future studies should aim at recruiting larger and comparable samples depending on the different comorbidities.

1.1. Subgroup Analysis BPD with and without a current PTSD

Sample Description

The sample of acute BPD patients was split in two groups depending on PTSD-co-diagnosis: BPD patients with a current PTSD (BPD-PTSD+) and BPD patients without a current PTSD (BPD-PTSD–). The subgroups did not differ in age or years of education (see table S1). BPD individuals with a current PTSD (BPD-PTSD+) scored significantly higher on all CTQ-subscales (see Table S1), with the exception of the subscale CTQ-'*emotional neglect*' than BPD subjects without a comorbid PTSD (BPD-PTSD–) (see Table S1). Both groups were comparable in psychopathology (for detailed description see Table S1).

Table S1. Sample description for acute BPD patients with (PTSD+) and without PTSD (PTSD–)

	PTSD– N=55		PTSD+ N=21			
	AM	SD	AM	SD	T	p
RSQ	15.54	5.41	14.72	5.29	.60	.55
BSL	1.89	.78	1.9	.60	-.04	.97
BDI	23.00	10.95	23.43	8.62	-.18	.86
Self-Esteem	9.45	5.38	12.1	13.83	-1.20	.23
CTQ-Total	57.57	15.16	72.89	19.35	-3.42	<.01
CTQ- *physical abuse*	7.57	3.68	10.95	5.53	-2.57	.02
CTQ- *physical neglect*	9.28	3.18	11.88	4.27	-2.81	<.01
CTQ- *emotional abuse*	16.26	5.01	18.86	4.71	-2.03	<.05
CTQ- *emotional neglect*	16.50	4.94	18.65	4.39	-1.69	.10
CTQ- *sexual abuse*	7.75	11.85	11.85	6.23	-2.72	.01
age	28.13	6.83	28.76	4.95	-.39	.70
years of education	11.87	1.52	11.95	1.53	-.20	.84

Covariation of RSQ with borderline symptom severity

To assess whether a current comorbid diagnosis of PTSD might have an impact on the covariation of rejection sensitivity and borderline symptoms, we run correlation analyses separately for BPD-PTSD+ and BPD-PTSD– -subjects. Rejection sensitivity only correlated significantly with BPD symptom severity (BSL) within the BPD-PTSD– -group (see Table S2). The strength of correlation did not differ significantly between the groups ($p > .1$).

Table S2: Pearson´s correlation coefficients between Rejection Sensitivity (RSQ), borderline symptom severity (BSL-23), childhood maltreatment (CTQ), self-esteem (SES) and depressive symptoms (BDI) for BPD subjects without a comorbid PTSD (BPD-PTSD–), with a comorbid PTSD (BPD-PTSD+)

	BSL		CTQ		SES		BDI	
	PTSD–	PTSD+	PTSD–	PTSD+	PTSD–	PTSD+	PTSD–	PTSD+
RSQ	.39**	.02	.15	.43(*)	-.40**	-.43(*)	.34*	-.26
BSL	-	-	-.01	.18	-.57***	-.20	.79***	.68**
CTQ	-	-	-	-	-.11	-.33	-.13	.06
SES	-	-	-	-	-	-	-.56***	-.08

(*)=p<.10, *=p<.05, **= p<.01, ***=p<.001

RSQ, childhood maltreatment, and borderline symptom severity

In the BPD-PTSD– -group rejection sensitivity was only linked to borderline symptom severity and depressive symptoms, but was not associated with childhood maltreatment as assessed with the CTQ. In the BPD-PTSD+ -group no association between rejection sensitivity, childhood maltreatment and BPD symptom severity could be found (see Table S2).

For explorative purposes, we additionally calculated correlations separately for each subscale of the CTQ (Table S3). In the subgroups only two significant correlations could be observed. In the BPD-PTSD+ -group CTQ-'sexual abuse' correlated positively with RSQ, but could not be linked to borderline symptom severity in this subgroup. In contrast, in the BPD-PTSD– -group CTQ-'sexual abuse' was not associated with RSQ but was negatively correlated with borderline symptom severity. RS and BSL scores did not correlate with any of the remaining CTQ subscales (see Table S3). Because of this lack in co-variation, no mediation analysis was calculated for the BPD subgroups.

Table S3: Pearson´s correlation coefficients between CTQ-subscales (emotional abuse: EA, emotional neglect: EN, physical abuse: PA, physical neglect: PN, sexual abuse: SA) and Rejection Sensitivity (RSQ), borderline symptom severity (BSL-23), self-esteem (SES) and depressive symptoms (BDI) for BPD subjects without a comorbid PTSD (BPD-PTSD–), with a comorbid PTSD (BPD-PTSD+)

	CTQ-EA		CTQ-EN		CTQ-PA		CTQ-PN		CTQ-SA	
	PTSD–	PTSD+	PTSD–	PTSD+	PTSD–	PTSD+	PTSD–	PTSD+	PTSD–	PTSD+
RSQ	.11	.19	.17	.15	.02	.26	.25(*)	.41(*)	.00	.57**
BSL	.18	.20	.15	-.01	-.15	.02	.01	.28	-.29*	.11
BDI	.04	.23	.01	.00	-.08	.01	-.05	.11	-.35*	-.09
SES	-.26(*)	-.14	-.19	-.24	.01	-.28	-.12	-.31	.20	-.23

(*)=p<.10, *=p<.05, **= p<.01, ***=p<.001

RSQ, self-esteem and borderline symptom severity

RSQ, SES, and BSL were only closely related within BPD-PTSD– -individuals (see Table S2). To test whether the association between RS and borderline symptom severity is mediated by self-esteem, we applied a hierarchical regression with a subsequent Sobel-z-test and a non-parametric bootstrapping method.

We run a hierarchical regression analyses for the BPD-PTSD–-individuals. We found a similar results pattern as already mentioned for the complete acute BPD group: (1) the BSL score was predicted by the RSQ, 2) self-esteem was predicted by the RSQ, and 3) the BSL score was predicted simultaneously by the RSQ and the SES). The RSQ score predicted both, the BSL score ($b=.06$, $t = 3.31$, $p <.01$) as well as the SES score ($b=-.39$, $t=-2.97$, $p <.01$). Taking both, the RSQ and the SES score simultaneously into account, only the SES significantly predicted Borderline symptom severity ($b=-.07$, $t =-3.88$, $p <.001$), while the prediction of the RSQ disappeared ($b=.03$, $t=1.95$, $p=.06$). The Sobel z-test examined a significant indirect effect of the RSQ on the BSL through the SES score ($z=2.31$, $p=.02$).

To account for violations of the normal distribution of rating scores in the BPD-PTSD– -group, a non-parametric bootstrapping method was additionally used to estimate the confidence interval of the indirect effect. The indirect effect was different from zero with a probability of 95%.

1.2. Subgroup Analysis BPD with and without a current Social Anxiety Disorder

Sample Description

The sample of acute BPD patients was split in two groups depending on Social Anxiety Disorder co-diagnosis: BPD patients with a current Social Anxiety Disorder (BPD-SAD+) and BPD patients without a current Social Anxiety Disorder (BPD-SAD–). The subgroups did not differ in age or years of education (see Table S4). BPD-SAD+ subjects reported significantly higher rejection sensitivity on the RSQ compared to the BPD-SAD– subject. Both groups were comparable regarding other psychopathology (for detailed description see Table S4).

Table S4. Sample description for acute BPD patients with (SAD+) and without current social anxiety disorder (SAD–)

	SAD–<br>N=53		SAD+<br>N=23			
	AM	SD	AM	SD	T	p
RSQ	14.39	5.27	17.46	5.03	-2.36	.02
BSL	1.89	.72	1.91	.78	-.13	.90
BDI	22.43	9.71	24.65	11.56	-.86	.39
Self-Esteem	11.21	9.62	7.87	5.07	1.57	.12
CTQ-Total	61.11	15.97	64.44	22.25	-.67	.51
CTQ- *physical abuse*	8.62	4.39	8.50	5.05	.10	.91
CTQ- *physical neglect*	9.84	3.48	10.55	4.27	-.72	.47
CTQ- *emotional abuse*	17.00	5.09	17.10	5.01	-.08	.94
CTQ- *emotional neglect*	17.04	4.85	17.37	4.99	-.25	.81
CTQ- *sexual abuse*	8.20	3.57	10.89	7.52	-1.50	.15
age	27.83	6.32	29.39	6.39	-.99	.33
years of education	11.87	1.52	11.95	1.53	-.74	.46

Covariation of RSQ with borderline symptom severity

To assess whether a current comorbid diagnosis of social anxiety disorder might have an impact on the covariation of rejection sensitivity and borderline symptoms, we run analyses for BPD subjects with an acute social anxiety disorder (BPD-SAD+) and without one (BPD-SAD–). Rejection sensitivity only correlated significantly with BPD symptom severity (BSL) within BPD-SAD+ group ($r = .58$; $p < .01$). The strength of correlation did not differ significantly between the groups ($p > .1$).

RSQ, childhood maltreatment, and borderline symptom severity

Neither rejection sensitivity nor BPD symptom severity could be linked to childhood maltreatment as assessed with the within the BPD subgroups BPD-SAD+ and BPD-SAD– (see Table S5).

For explorative purposes, we additionally calculated correlations separately for each subscale of the CTQ. In the BPD subgroups, RSQ as well as BSL did not correlate with any of the CTQ subscales (see Table S6). Because of this lack in co-variation, no mediation analysis was calculated for the BPD subjects with and without SAD.

RSQ, self-esteem and borderline symptom severity

RSQ, SES, and BSL were only closely related within BPD-SAD+ individuals (see Table S5). To test whether the association between RS and borderline symptom severity is mediated by self-esteem in the BPD-SAD+ subgroup, we applied a hierarchical regression with a subsequent Sobel-z-test and a non-parametric bootstrapping method.

We found a similar results pattern for BPD subjects with a comorbid SAD as already mentioned for the complete acute BPD group: (1) the BSL score was predicted by the RSQ, 2) self-esteem was predicted by the RSQ, and 3) the BSL score was predicted simultaneously by the RSQ and the SES). The RSQ score predicted both, the BSL score ($b=.09$, $t=3.28$, $p <.01$) as well as the SES score ($b=-.55$ $t=-3.01$, $p <.01$). Taking both, the RSQ and the SES scores simultaneously into account, only the SES significantly predicted Borderline symptom severity ($b=-.07$ $t=-2.50$, $p=.02$), while the prediction of the RSQ disappears again ($b= .05$

t=1.69, p=.11). There was a trend for a significant indirect effect of the RSQ on the BSL through the SES score (z=1.86, p=.06).

To account for violations of the normal distribution of rating scores in BPD-SAD+ - individuals, a non-parametric bootstrapping method was additionally used to estimate the confidence interval of the indirect effect. The indirect effect was different from zero with a probability of 95% for the BPD-SAD+ group.

Table S5: Pearson´s correlation coefficients between Rejection Sensitivity (RSQ), borderline symptom severity (BSL-23), childhood maltreatment (CTQ), self-esteem (SES) and depressive symptoms (BDI) for BPD subjects without a comorbid SAD (BPD-SAD–), with a comorbid SAD (BPD-SAD+)

	BSL		CTQ		SES		BDI	
	SAD–	SAD+	SAD–	SAD+	SAD–	SAD+	SAD–	SAD+
RSQ	.20	.58**	.18	.21	-.32*	-.55**	.07	.42*
BSL			.02	.08	-.28*	-.65**	.80***	.73***
CTQ					-.14	-.16	.12	-.36
SES							-.21	-.59**

(*)=p<.10, *=p<.05, **= p<.01, ***=p<.001

Table S6: Pearson´s correlation coefficients between CTQ-subscales (emotional abuse: EA, emotional neglect: EN, physical abuse: PA, physical neglect: PN, sexual abuse: SA) and Rejection Sensitivity (RSQ), borderline symptom severity (BSL-23), self-esteem (SES) and depressive symptoms (BDI) for BPD subjects without a comorbid SAD (BPD-SAD–), with a comorbid SAD (BPD-SAD+)

	CTQ-EA		CTQ-EN		CTQ-PA		CTQ-PN		CTQ-SA	
	SAD–	SAD+	SAD–	SAD+	SAD–	SAD+	SAD–	SAD+	SAD–	SAD+
RSQ	.08	.21	.16	.09	.04	.20	.27(*)	.24	.11	.18
BSL	.12	.30	.10	.16	-.02	-.22	-.04	.31	-.10	-.20
BDI	.09	.09	.16	-.31	.18	-.45*	.09	-.16	-.02	-.50*
SES	-.11	-.33	-.16	-.12	-.14	-.02	-.13	-.31	.04	.06

(*)=p<.10, *=p<.05
